# Natural Language AI Models and Pediatric Type 1 Diabetes: Can Chatbots Help With Diabetes Self-Management and Patient Education?

**DOI:** 10.2196/76986

**Published:** 2025-11-07

**Authors:** Flavia Voiculescu, Paul Darvasi, Esli Osmanlliu, Preetha Krishnamoorthy, Angeliki Makri

**Affiliations:** 1 Centre for Outcomes Research and Evaluation (CORE), Research Institute of the McGill University Health Center, Montreal, QC, Canada; 2 Department of Curriculum, Teaching and Learning, University of Toronto, Toronto, ON, Canada; 3 Division of Emergency Medicine, Department of Pediatrics, Montreal Children’s Hospital, McGill University Health Centre, Montreal, QC, Canada; 4 Division of Endocrinology and Metabolism, Department of Pediatrics, Montreal Children's Hospital, McGill University Health Centre, A04.6314, 1001 Decarie Boulevard, Montreal, QC, H4A 3J1, Canada, 1 5144124400

**Keywords:** type 1 diabetes, chatbots, artificial intelligence, patient education, diabetes self-management

## Abstract

Diabetes self-management plays a major role in controlling blood sugar levels and avoiding chronic complications. In this report, we investigate the strengths and limitations of artificial intelligence chatbots in supporting patients with type 1 diabetes and their families. With the growing accessibility of these constantly evolving tools, front-line providers must advocate for their responsible use.

## Introduction

Type 1 diabetes (T1D) is one of the most common chronic diseases of childhood with significant morbidity and mortality. High-level evidence supports the optimization of blood sugar control to prevent chronic complications [1]. Even with remarkable advances in diabetes technology, such as continuous glucose monitoring systems and automated insulin delivery pumps, a majority of patients fail to meet glycemic targets [2].

Diabetes self-management and education play a major role in glycemic control. However, several challenges limit the potential of traditional educational models: shortages of diabetes educators, reduced access to care for some patient groups (exacerbated by the recent pandemic), and inherent limitations to addressing patients’ and caregivers’ individual needs.

Chatbots are tools powered by artificial intelligence (AI) that have shown promise in many aspects [3]. They are computer programs designed to respond to questions in natural language as if they were a real person. It is anticipated that such tools will be increasingly used by patients and their families because of their ease of access. Clinicians nonetheless remain a key source for trusted health information, and pediatricians and other front-line health care providers must understand the strengths and limitations of such tools.

A recent paper in *Diabetes Care* [4] noted strengths and inaccuracies of the recent GPT-4 model developed by OpenAI when asked questions on diet/exercise, glycemic management, and insulin storage and administration in adult patients. Children with T1D have unique needs regarding self-management when compared to adults. In this brief report, we sought to evaluate the strengths and weaknesses of an AI tool for pediatric diabetes management.

## Methods

We asked a multidisciplinary team at the Pediatric Diabetes Clinic of the Montreal Children’s Hospital at McGill University Health Centre, a tertiary care center caring for 900 pediatric patients with T1D, to help craft a comprehensive list of questions regarding education and self-management of children with T1D spanning 4 key categories (Textbox 1). The multidisciplinary team included a pediatric endocrinologist, a pediatric emergency room physician, a medical student with previous experience in pediatric diabetes research, 3 certified diabetes educators, and 1 parent partner, all affiliated with the Montreal Children’s Hospital.

The questions were asked in both French and English to the chatbot, and its answers were evaluated qualitatively based on whether they reflected what is routinely taught to families and whether the answers were complete, accurate, and correct.

Textbox 1. **Questions asked to ChatGPT with GPT-4.**

**New diagnosis**

1. My child was just diagnosed with T1D. What is this? I have only heard of diabetes in older obese adults.
2. Will my child have any restrictions in his life?
3. When will my child be ready to go back to school? What should I say to the school teacher?
4. Can you recommend reliable websites where I can read more about T1D?

**Recognizing and managing hypo- and hyperglycemia**

1. What is the blood sugar level that the doctors kept mentioning?
2. What is a normal blood sugar?
3. What symptoms will my child have if his/her blood sugar is high or low?
4. What do I do if my child’s blood sugar is high or low?
5. My child is vomiting and his blood sugar is high. Should I bring him to the ER?

**Glucometers and insulin**

1. My child’s doctor and nurse showed to me how to use a glucometer but I can not remember the exact steps. Can you give me step by step instructions?
2. My child’s doctor and nurse gave me 2 different insulins; a long acting and a short acting. What is the difference?
3. My child’s doctor and nurse showed me how to use an insulin pen but I cannot remember the exact steps. Can you give me step by step instructions?
4. Which body parts should I use to do the insulin injections?

**Newer diabetes technologies**

1. My child’s doctor and nurse mentioned continuous glucose monitors and insulin pumps. What are these? Are they recommended for children with T1D?
2. How often do I need to change the CGM and the pump?
3. How do I interpret the different metrics on a CGM? What is the goal target in range?
4. What type of insulin is given in an insulin pump?

## Results

Textbox 2 compiles the feedback given by the multidisciplinary diabetes team, highlighting strengths and limitations of ChatGPT for diabetes self-management and patient education. We found that the answers provided by ChatGPT had the same limitations in both French and English.

Textbox 2. **Feedback provided by the multidisciplinary diabetes team on ChatGPT’s answers.**

**Strengths**

- Conversation tone imitated a real human-to-human interaction
- Clear instructions in point form
- Easily understood and mostly accurate responses from multiple trustworthy online sources
- No medical jargon
- Most answers safely highlighted the importance of remaining in contact with the child’s health care provider
- Generally useful information, available 24-7
- Potential for immediate translation to support families who face language barriers

**Limitations**

- New diagnosis
  - No reference to the International Society for Pediatric and Adolescent Diabetes, which provides specific guidelines for children, such as a target blood glucose range of 70-180 mg/dl (4-10 mmol/L)
- Recognizing and managing hypo- or hyperglycemia
  - No mention of home ketone testing in the management of hyperglycemia, a critical step in assessing the risk of diabetic ketoacidosis while the child is still at home
  - No mention that the amount of carbohydrates used to treat hypoglycemia depends on the child’s weight; for example giving 15 g of carbohydrates to a 20 kg child would cause rebound hyperglycemia, while 5 g of carbohydrates to an adolescent would not be enough; also, the type of insulin therapy influences how a low blood glucose is treated; for example, if the child is on standard pump therapy and has a glucose level <3 mmol/L, we recommend suspending insulin delivery until glucose >4 mmol/L
- Glucometers and insulin analogs
  - It would be useful if images accompanied the step-by-step instructions on how to use a glucometer, the insulin pen, and the body parts where insulin injections can be self-administered.
  - No mention of admelog (insulin lispro) or trurapi (insulin aspart), insulin products approved by Health Canada. No mention of Tresiba, a newer long-acting insulin often used to provide more flexibility.
- Newer diabetes technologies
  - Blood glucose is provided only in mg/dl. ChatGPT did not provide the international consensus guidelines on time above range (<25%) and time below range (<4%).
  - Incorrect information that insulin pumps only give short- or rapid-acting insulin. Long-acting insulin is not necessary as the pump delivers tiny amounts of insulin every few minutes.

## Discussion

When asked common questions on T1D, ChatGPT provided useful answers, though they were at times lengthy and lacked pediatric-specific details. It used clear terminology that had the potential for immediate translation to different languages. One important deficiency was noted regarding counseling for hyperglycemia, as it omitted the need for ketone measurement at home. The chatbot also provided inaccurate information on the type of insulin delivered by pumps (Textbox 2).

AI chatbots for diabetes education of patients and families can free precious health care provider time, allowing clinicians to support patients with the greatest need for more frequent human touchpoints. A human-AI interaction that integrates safeguards such as easily interpretable care algorithms can thus enable more frequent technology-enabled contact.

To understand the limitations of AI tools in diabetes education, we need to consider the way that they are developed and trained. They use an extraordinarily large text database from the internet and generate answers based on the likelihood of phrases following each other. They are not trained on databases specific to medical information and are not capable of evaluating the reliability of the answers they provide. Thus, they are prone to the phenomenon of “hallucination”: answers that sound reasonable but are factually incorrect [5].

As with any new technology in health care, it is important to consider ChatGPT’s strengths, limitations, and ethical implications prior to implementing its use in routine clinical practice. It is essential to ensure that AI-based resources are easily accessible to our patients to prevent worsening existing health care disparities. Moreover, transparency in how information is generated and communicated is critical, so that patients can interpret responses with appropriate caution. With the growing ease of access to these constantly evolving tools, front-line providers must advocate for use that is responsible and promotes value-based care [6].

## Abbreviations

AI: artificial intelligence
T1D: type 1 diabetes
